# Initial Psychometric Properties of 7 NeuroUX Remote Ecological Momentary Cognitive Tests Among People With Bipolar Disorder: Validation Study

**DOI:** 10.2196/36665

**Published:** 2022-07-29

**Authors:** Raeanne C Moore, Emma M Parrish, Ryan Van Patten, Emily Paolillo, Tess F Filip, Jessica Bomyea, Derek Lomas, Elizabeth W Twamley, Lisa T Eyler, Colin A Depp

**Affiliations:** 1 Department of Psychiatry University of California San Diego San Diego, CA United States; 2 SDSU/UC San Diego Joint Doctoral Program in Clinical Psychology San Diego, CA United States; 3 Psychiatry and Human Behavior Brown University Providence, RI United States; 4 Providence VA Medical Center Providence, RI United States; 5 VA San Diego Healthcare System San Diego, CA United States; 6 Industrial Design Engineering Delft University of Technology Delft Netherlands

**Keywords:** neuropsychology, mobile health, ambulatory assessment, ecological momentary assessment, practice effects, validity, testing, serious mental illness, mobile phone

## Abstract

**Background:**

As smartphone technology has become nearly ubiquitous, there is a growing body of literature suggesting that ecological momentary cognitive testing (EMCT) offers advantages over traditional pen-and-paper psychological assessment. We introduce a newly developed platform for the self-administration of cognitive tests in ecologically valid ways.

**Objective:**

The aim of this study is to develop a Health Insurance Portability and Accountability Act–compliant EMCT smartphone-based platform for the frequent and repeated testing of cognitive abilities in everyday life. This study examines the psychometric properties of 7 mobile cognitive tests covering domains of processing speed, visual working memory, recognition memory, and response inhibition within our platform among persons with and without bipolar disorder (BD). Ultimately, if shown to have adequate psychometric properties, EMCTs may be useful in research on BD and other neurological and psychiatric illnesses.

**Methods:**

A total of 45 persons with BD and 21 demographically comparable healthy volunteer participants (aged 18-65 years) completed smartphone-based EMCTs 3 times daily for 14 days. Each EMCT session lasted approximately 1.5 minutes. Only 2 to 3 tests were administered in any given session, no test was administered more than once per day, and alternate test versions were administered in each session.

**Results:**

The mean adherence to the EMCT protocol was 69.7% (SD 20.5%), resulting in 3965 valid and complete tests across the full sample. Participants were significantly more likely to miss tests on later versus earlier study days. Adherence did not differ by diagnostic status, suggesting that BD does not interfere with EMCT participation. In most tests, age and education were related to EMCT performance in expected directions. The average performances on most EMCTs were moderately to strongly correlated with the National Institutes of Health Toolbox Cognition Battery. Practice effects were observed in 5 tests, with significant differences in practice effects by BD status in 3 tests.

**Conclusions:**

Although additional reliability and validity data are needed, this study provides initial psychometric support for EMCTs in the assessment of cognitive performance in real-world contexts in BD.

## Introduction

### Background

Advances in digital technology and the increasing ubiquity of both internet access [1] and mobile phones [2] are changing the way in which social, emotional, and cognitive states are measured. Ecological momentary assessments (EMAs) [3] deliver brief, repeated, self-reported surveys to examinees as they go about their daily lives. EMAs are designed to capture naturalistic fluctuations in psychological and physiological states in real time without relying on retrospective recall. A growing body of literature supports the utility of EMAs in a variety of populations [4-9], and EMAs have shown greater sensitivity to psychological distress compared with single-time psychological assessments. For example, Moore et al [10] reported that EMA measures were more sensitive to subtle reductions in depression and anxiety after a mindfulness intervention than identical paper-pencil instruments measuring the same constructs.

EMA methods are increasingly being used to assess cognitive performance through brief, repeated neuropsychological tasks. In this context, we refer to the concurrent administration of EMA questions and mobile cognitive tests as ecological momentary cognitive testing (EMCT). Owing to the dynamic nature of cognitive processes with influence from a variety of situational factors [11], single-administration assessment data from traditional in-person cognitive testing are vulnerable to the acute impact of confounds such as sleep deprivation and mood instability. In contrast, EMCT data can be aggregated across multiple testing sessions, thereby potentially reducing the influence of intraindividual error from extraneous variables [12-14]. In addition, EMCT data can be used to directly examine intraindividual variability in cognitive performance across time and contexts. Overall, the delivery of EMCT measures to individuals in real-world environments likely improves ecological validity over controlled laboratory or clinic-based evaluations [12,13,15-17]. In contrast, an advantage of traditional neuropsychological testing over EMCT is the precise control over the environment, allowing for optimization of performance and in-depth measurement of cognitive functioning. Accordingly, EMCT is intended to augment rather than replace comprehensive neuropsychological evaluations [14,18].

Bipolar disorder (BD) may be a particularly appropriate condition for which to implement EMCTs given the symptom fluctuation that characterizes the disorder [19]. Moreover, cognitive sequelae of BD are well recognized [20,21], and cognitive performance is more variable in patients with BD than in controls [21]. Importantly, symptom severity and variability in BD do not prevent the collection of EMA data as multiple studies of psychological constructs have been successfully carried out in patients with BD [22-26].

### Objectives

The goal of this study was to investigate the psychometric properties of 7 newly developed mobile cognitive tests administered within an EMCT platform (NeuroUX) in adults with BD and healthy comparison participants. These tests were designed to measure processing speed, working memory, visual memory, verbal recognition memory, reasoning, and inhibitory control. We examined adherence, practice and fatigue effects, intraindividual variability, reliability and validity metrics, and performance differences based on BD status.

EMA adherence in BD, depression, and schizophrenia has varied across studies, with most reporting rates of at least 65% and sometimes >90% [24,27-31]. This study included both EMA and mobile cognitive testing (EMCT) as opposed to EMA only, which could have affected the participants’ engagement. Meta-analytic studies of compliance with EMA protocols [30,32,33] range from median adherence rates of 75% to 80%. It is important to note that approximately 10% of participants in EMA studies have significantly lower adherence (approximately 45% [30]), which lowers the mean adherence rates. We aimed for a benchmark of 70% mean adherence in our sample given the extra time commitment needed to complete EMA surveys and mobile cognitive tests in each session.

Phone type differences were examined to address device variability, such as differences in response time latencies that have the potential to affect the recorded test performance [34,35]. To examine convergent and discriminant validity, we also compared aggregate mean scores on the mobile cognitive tests with the National Institutes of Health Toolbox Cognition Battery (NIH-TB-CB), iPad version [36,37], and examined intraindividual variability between groups. We hypothesized that (1) there would be adequate adherence to the EMCT protocol and small practice effects across the full sample, and these metrics would not vary by diagnostic status; (2) EMCT scores would be moderately associated with laboratory-based neuropsychological test scores, and these associations would be strongest among clinical tests assessing the same or similar cognitive processes—specifically, that our mobile cognitive tests of processing speed (Matching Pair, Odd One Out [time to complete], and Quick Tap 1) would be related to global cognition (ie, the National Institutes of Health Toolbox [NIH-TB] Fluid Composite) and to all the individual cognitive domains; that our mobile cognitive tests of working memory (Memory Matrix, Odd One Out [total score], and CopyKat) would be related to global cognition and tests in the domains of working memory, processing speed, and executive function; that our recognition memory mobile cognitive test (Mobile Variable Difficulty List Memory Test [VLMT]) would be related to the NIH-TB memory test and, although less strongly, to global cognition; and that our response inhibition mobile cognitive test (Quick Tap 2) would be related to laboratory-based tests of attention processing speed, working memory, and executive function. We also hypothesized that poorer performance on the mobile cognitive tests would be associated with a diagnosis of BD, older age, and fewer years of education and that intraindividual variability would be higher in the BD group than in the healthy volunteer group.

## Methods

### Recruitment

Participants were recruited through flyers and web-based recruitment portals and were either individuals with BD (n=45) or demographically comparable healthy volunteers (n=21). The inclusion criteria were as follows: (1) a diagnosis of BD on the MINI International Neuropsychiatric Interview version 6.0.0 [38] or no psychiatric diagnoses for healthy volunteers, (2) outpatient treatment status for the BD group, (3) age between 18 and 65 years, (4) fluency in English, (5) capacity to provide written informed consent, and (6) not being on conservatorship. The exclusion criteria were as follows: (1) history of neurological disorder or head trauma with loss of consciousness for >15 minutes, (2) sensory impairment, (3) substance use disorder in the previous 3 months (excluding cannabis and tobacco), (4) severe manic symptoms as measured by a score >20 on the Young Mania Rating Scale [39] or severe depressive symptoms as measured by a score >30 the Montgomery-Asberg Depression Rating Scale for the BD group [40], (5) ideation score type 3 or higher on the Columbia Suicide Severity Rating Scale (C-SSRS) [41] in the previous month, and (6) concurrent enrollment in another research study.

### Ethics Approval

This study was approved by the University of California San Diego Institutional Review Board (protocol #172120), and all participants provided written informed consent and demonstrated capacity to consent based on a brief screening measure [42]. At the screening visit, the participants completed interview-rated symptom measures and the C-SSRS.

### Procedure

At the baseline visit, the participants completed self-report questionnaires and laboratory-based neuropsychological performance tests, including the NIH-TB-CB and Delis-Kaplan Executive Function System (D-KEFS) Color-Word Inference Test. The NIH-TB-CB iPad version was used, which includes a mix of self-administered and examiner-assisted–administered tests. An examiner is required to be present for the full battery, which takes approximately 30 to 45 minutes to administer. The D-KEFS Color-Word Inference Test is examiner-administered and takes approximately 3 minutes to administer. The participants could either use their own smartphones or borrow a study-provided Apple iPhone 7 for home-based EMCT. The participants were trained on the EMCT protocol, and all of them (66/66, 100%) completed a laboratory-based session of the 7 mobile cognitive tests with time for technical questions and troubleshooting. Participants also received an EMCT operating manual, which we developed for this study. The manual included information on when to expect the alerts to take the mobile cognitive tests, directions for how to complete the EMA surveys and mobile cognitive tests, important tips and reminders (eg, reminder to charge their smartphone nightly), troubleshooting tips, and frequently asked questions.

The 14-day EMCT protocol began the day following the baseline visit and consisted of 3 SMS text message notifications per day, with each notification including a link to an EMA survey and 2 to 3 mobile cognitive tests (Table 1). The timing of these testing windows was adjusted according to each person’s sleep and wake schedule, and there was a 2-hour minimum between each testing session; links were active for 1 hour after the SMS text message notification. Each of the 7 mobile cognitive tests was administered 9 times over the 14-day testing period (with the exception of the Mobile VLMT, which was administered daily), and the order was counterbalanced to ensure that each test was administered evenly across the morning, midday, and early evening. Different versions of the tasks were administered at each time point. No identifying information was affiliated with the EMCT platform, and deidentified data were instantly uploaded to Amazon Web Services (Health Insurance Portability and Accountability Act–compliant).

**Table 1:** Protocol for mobile cognitive testing administration.

Mobile cognitive test	Study day													
	1	2	3	4	5	6	7	8	9	10	11	12	13	14
Matching Pair	MA^a^	EE^b^	EE	EE	MA	—^c^	—	—	MD^d^	—	—	MA	MD	MD
Memory Matrix	—	MD	—	MA	MD	MA	EE	—	EE	EE	—	EE	—	MA
Odd One Out	—	MA	—	MD	EE	—	—	EE	MA	MA	MD	MA	—	EE
VLMT^e^ recall	EE	MA	MA	EE	MA	MD	MD	MD	EE	MA	EE	MD	MD	EE
VLMT recognition^f^	EE	MA, MD	MA, MD	EE	MA, MD	MD, EE	MD	MD, EE	EE	MA, MD	EE	MD, EE	MD, EE	EE
Quick Tap 1	EE	—	MA	—	—	MD	MD	MD	—	EE	MA	MD	—	MA
Quick Tap 2	EE	—	MA	—	—	MD	MD	MD	—	EE	MA	MD	—	MA
CopyKat	MD	—	MD	—	—	EE	MA	MA	MD	—	EE	EE	MA	—

^a^MA: morning administration.

^b^EE: early evening administration.

^c^Empty cells indicate that a session was not scheduled at that day and time.

^d^MD: midday administration.

^e^VLMT: Variable Difficulty List Memory Test.

^f^The VLMT recognition was sometimes administered at 2 time points on the same day.

The study staff contacted participants by telephone on the first day of the at-home

protocol, as well as if participants missed >3 surveys in a row, to increase adherence and help troubleshoot any problems. Participants were provided with study staff information, who were available Monday to Friday (and often on Saturdays) to respond to questions and help troubleshoot problems. Following the 14-day EMCT data collection period, participants returned to the laboratory to complete the C-SSRS again and a study feedback questionnaire as well as to return the study smartphone (if one was borrowed). Participants were compensated for all study visits and were given a bonus compensation of US $1 for each EMCT session completed.

### Measures

#### Baseline Symptom Measures

Participants with BD were assessed for depression using the interview-rated Montgomery-Asberg Depression Rating Scale, with total scores ranging from 0 to 60 and higher scores indicating greater severity [40]. Symptoms of mania were assessed using the Young Mania Rating Scale, with a total score of 0 to 60 and higher scores indicating greater severity [39].

#### Baseline Cognitive Measures

The NIH-TB-CB [37] consists of 7 tests designed to measure language (Oral Reading Recognition), episodic memory (Picture Sequence Memory Test), working memory (List Sorting Working Memory Test), attention (Flanker), executive functioning (Flanker and Dimensional Change Card Sort Test), and processing speed (Pattern Comparison Processing Speed Test). Scores from the attention, episodic memory, working memory, executive functioning, and processing speed tests are averaged to create a Fluid Cognition score. The NIH-TB-CB also includes language tests (receptive vocabulary and reading) as an estimate of premorbid functioning and education (Crystallized Intelligence score). The NIH-TB-CB was normed in a large sample representative of the US population aged 3 to 85 years. The age-corrected standard scores were used in this study [43]. We chose not to use demographically adjusted T-scores to conduct more direct comparisons between NIH-TB-CB test scores and our mobile cognitive tests.

#### EMCT Platform

The development of our EMCT platform was supported in part by the National Institute of Mental Health. The mobile cognitive tests were designed using a human-centered approach and gamified to increase engagement [44]. Test stimuli differed at each administration, thus serving as alternate forms. The platform was first beta-tested in 10 healthy adults across their life span, and modifications were made based on user feedback. A total of 7 mobile cognitive tests were initially developed and tested in this study. Refer to Table 2 for a list of the mobile cognitive tests, the cognitive domains assessed, completion times, and screenshots.

**Table 2:** Mobile cognitive tests.

Mobile cognitive test	Cognitive domain assessed	Time to complete	Screenshot of task
Matching Pair	Processing speed	90 seconds (fixed time)	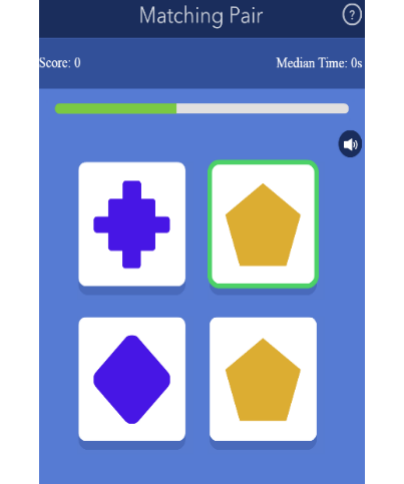
Memory Matrix	Visual working memory	Variable; 3 trials; approximately 1 to 2 minutes (mean completion time 1.5, SD 1.4 minutes)	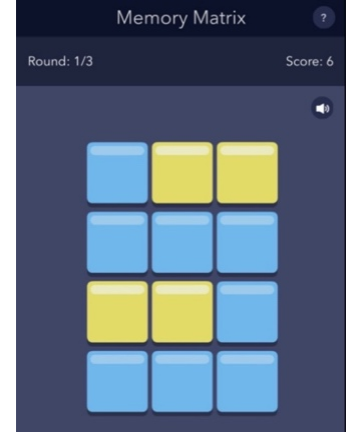
Odd One Out	Visual working memory (primary); processing speed (secondary)	Variable; 9 trials; approximately 1 minute (mean completion time 0.75, SD 0.57 minutes)	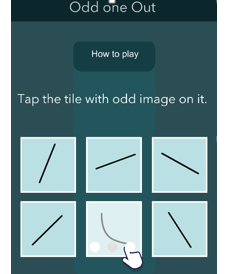
VLMT^a^	Recognition memory	30 seconds for list presentation (fixed time)	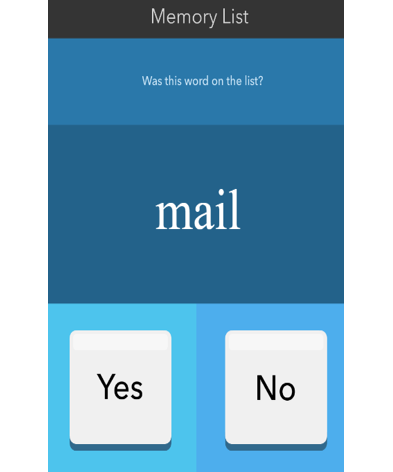
Quick Tap 1	Processing speed	Variable; 12 trials; approximately 1 minute (mean completion time 60.8, SD 64.6 seconds)	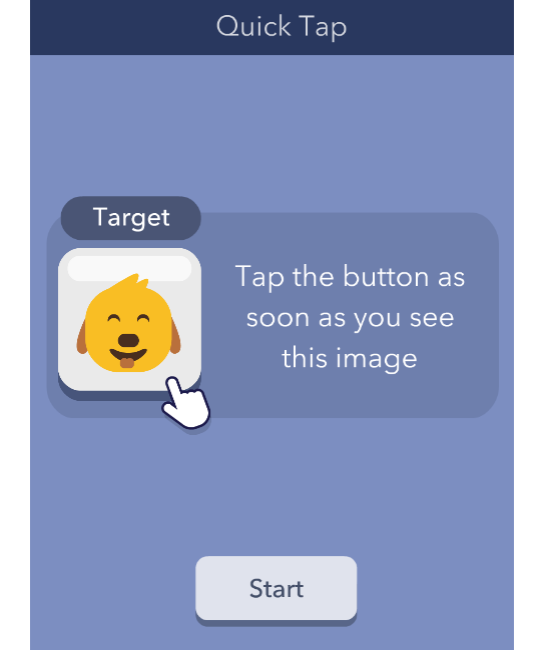
Quick Tap 2	Response inhibition	Variable; 12 trials; approximately 1 minute (mean completion time 66.8, SD 20.6 seconds)	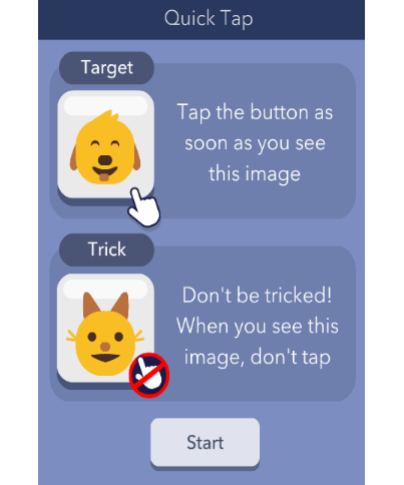
CopyKat	Visual working memory	Variable; 3 trials; approximately 2 to 3 minutes (mean completion time 2.7, SD 4.0 minutes)	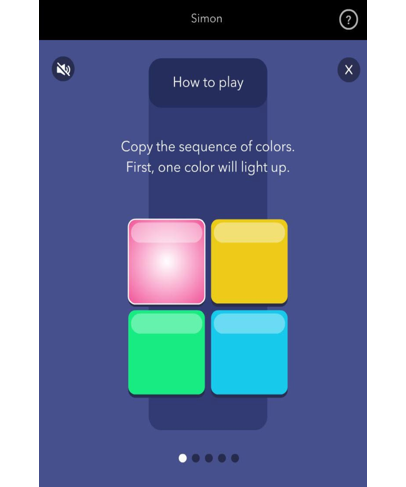

^a^VLMT: Variable Difficulty List Memory Test.

#### Mobile Cognitive Test Descriptions

##### Matching Pair

The cognitive domain assessed is processing speed, and the time to complete is 90 seconds. In Matching Pair, participants are presented with a matrix containing tiles of varying colors and shapes. This tile matrix starts as a 2×2 grid (4 tiles) and gradually increases to a maximum grid of 4×4 (16 tiles). Participants select the 2 tiles that match in color as quickly as possible using 1 finger. Scoring is a weighted accuracy score calculated according to the grid size shown. Faster response times are associated with the possibility of higher scores. The grid size is multiplied (eg, 4 × 4 = 16) and added to the running score of the previous correct trial. For example, for a correct trial with a grid size of 3×3 and a previous trial score of 246, the trial score would be calculated as follows: 3 × 3 = 9 and 9 + 246 = a trial score of 255. If a pair is incorrectly selected, the score does not change. The reaction time for each response is also recorded.

##### Memory Matrix

The cognitive domain assessed is visual working memory, and the time to complete is variable (3 trials for approximately 1 to 2 minutes total; mean completion time 1.5, SD 1.4 minutes). In Memory Matrix, participants are presented with a matrix of tiles starting with 2×2 (4 tiles), which gradually increases to a maximum of 7×7 (49 tiles). A pattern of contrasting color tiles is presented, and participants are asked to learn the location of these highlighted tiles. The contrasting pattern disappears after 1.5 seconds, and participants touch the tiles that were previously presented. This sequence begins with 1 highlighted tile, and based on performance, it can increase to a maximum of 11 tiles. The number of highlighted tiles increases by 1 for each correct response and decreases by 1 for each incorrect response. The task terminates when the participant makes 3 incorrect responses. For each correct response, the number of highlighted tiles is added to the previous correct score. For example, if the previous trial score was 12 and a correct response is given in a trial with 4 highlighted cells, this trial’s new score would be calculated as 16. The score does not change if a trial response is incorrect. Reaction time is also recorded.

##### Odd One Out

The cognitive domain assessed is visual working memory (primary) and processing speed (secondary), and the time to complete is variable (9 trials that take approximately 1 minute; mean completion time 0.75 seconds, SD 0.57 minutes). In Odd One Out, the participants are presented with 6 symbols and identify which symbol differs from the others as quickly as possible. For example, a trial may have 5 pictures of squares and 1 picture of a rectangle. Participants must identify the rectangle as the correct choice. Each administration contains 9 trials. The task is scored by adding the number of correct responses. This total correct response score is recorded as a measure of working memory. The reaction time in seconds for each trial is also recorded as a secondary domain.

##### Mobile VLMT

The cognitive domain assessed is recognition memory, and the time to complete is approximately 2.5 minutes in total (approximately 1.5 minutes for short delay, which includes word list presentation, and 1 minute for long delay). Participants are presented with a list of 12 words and given 30 seconds to learn the words. The list is then removed, and participants complete a distractor task (ie, one of the other mobile cognitive tests). Next, participants are presented with 24 words, which are a mix of the 12 target words and 12 foil words in a recognition memory paradigm. Words are presented one by one, and participants indicate whether the word was on the original list (*yes* or *no*). A total of 14 different lists were presented over the protocol period, and each list was presented only once during each mobile cognitive testing session. The development of the word lists has been described in previous publications [15,45]. The word list recognition trial was always completed within the same survey session (short delay). In addition, 9 times over the 14 days, participants completed a second recognition trial of the list during the subsequent survey as a measure of delayed recognition. This second list recognition task (long delay) always occurred on the same day as the list presentation. The overall score is calculated as a sum of the number of target words correctly identified and the number of foil words correctly rejected, with a total possible score of 24. A modified version of the VLMT in a different sample of persons with serious mental illness was described by Parrish et al [15].

##### Quick Tap 1

The cognitive domain assessed is processing speed, and the time to complete is approximately 1 minute in total for 12 trials. In Quick Tap 1, participants are first presented with a gray square containing an image (eg, a cartoon dog) that is indicated as the *target*. Participants are asked to tap the target image as quickly as possible when it appears. At the start of a trial, the gray square says, *Wait for the target*. Then, the target image replaces this text in a randomly generated time interval of 1 to 5 seconds, and participants are asked to tap the target as quickly as they can. If the image is not selected, the trial times out 2 seconds after the target is displayed, and the response is marked as incorrect. Similarly, if the gray square is pressed before the target is shown, the response is incorrect, and the next trial begins. Each session contains 12 trials, and each session contains a different target image. This task is scored by averaging the reaction time of the correctly answered trials in seconds.

##### Quick Tap 2

The cognitive domain assessed is response inhibition, and the time to complete is approximately 1 minute in total for 12 trials. Quick Tap 2 is similar to Quick Tap 1 except that, in some instances, a foil or *trick* (eg, cartoon picture of a cat) image is presented instead of the target image. This foil image, although different, appears similar to the target image (eg, a cartoon boy smiling vs a cartoon boy with his tongue out), and both the target and trick are identified in the instructions before beginning the session. Each session contains a different target and trick combination. Participants respond by tapping the target when it appears as quickly as possible; they are instructed not to tap the foil. Similar to Quick Tap 1, either image is randomly presented within 1 to 5 seconds in each trial, and the next trial begins either immediately after the image is pressed or after 2 seconds of presentation of the image if the image is not selected. The probability of foil presentation for each trial is randomly generated and ranges from 30% to 60%. Quick Tap 2 always immediately follows Quick Tap 1. Each session contains 12 trials. Both Quick Tap 1 and Quick Tap 2 were presented 9 times each over the 14-day protocol period. For each correct response, either correctly tapping the target or not tapping the trick, the total score increases by 1 for a maximum possible score of 12. Reaction time is also recorded.

##### CopyKat

The cognitive domain assessed is visual working memory, and the time to complete is variable (3 trials that take approximately 2 to 3 minutes; mean completion time 2.7 minutes). Similar to the popular electronic game Simon, the participants are presented with a 2×2 matrix of colored tiles in a fixed position: red, yellow, blue, and green. The tiles briefly light up in a random order, and participants are asked to replicate the pattern by pressing on the colored tiles in the correct order. The number of tiles that light up begins at 1 and increases by 1 with each correct response. For a correct response, the next trial contains the same pattern as the previous trial plus 1 additional highlighted tile. When an incorrect response is made, the same sequence is presented again. Similarly, if no response is made after 20 seconds, the trial is marked as incorrect. There is no upper limit on the maximum number of tiles. The session ends after 3 incorrect responses. The task is scored by summing the number of correct trials. An additional task feature is that each color plays a distinguishable tone when highlighted if the phone volume is turned on.

### Statistical Analyses

To compare baseline demographics and clinical characteristics by diagnostic status, independent 2-tailed *t* tests or chi-square tests were used for continuous and categorical variables, respectively. Adherence was examined by calculating the percentage of completed tests for each participant. Average adherence was compared by diagnostic status using independent *t* tests. To understand what we call *fatigue effects* (ie, the likelihood of missing a test), a mixed effect logistic regression was used to examine whether the participants were more likely to miss a mobile cognitive test on later versus earlier study days. An interaction between diagnostic status and study day was included to examine whether fatigue effects differed by diagnosis. The scores on 71% (5/7) of the tests were normally distributed (Multimedia Appendix 1). Odd One Out (total score) had a restricted range of performance, approaching a ceiling effect. Quick Tap 2 had 4 outliers, resulting in a significant skew. We chose not to transform these raw data into analyses.

We created a composite score for the mobile cognitive tests excluding the Odd One Out total score and Quick Tap 2 owing to ceiling effects. To create this composite score, we first log-transformed variables that were positively skewed (CopyKat time, Odd One Out time, Quick Tap 1 average time, and CopyKat total) and squared negatively skewed variables (Matching Pair, Memory Matrix, and VLMT) to normalize them before creating the composite. We then created standardized *Z* scores from these variables, and these standardized *Z* scores were aggregated by participant and combined to create the composite score.

The intraclass correlation coefficients by group are presented in Multimedia Appendix 2. Intraindividual variability was calculated using the mean square of successive differences, which is the sum of the squared differences between 2 consecutive observations divided by 2 times the number of observations minus 1. An independent sample *t* test was used to determine if intraindividual variability differed between participants with BD and healthy volunteers. Performance on each mobile cognitive test was aggregated within each participant across all administrations to examine the average differences in performance by diagnostic group, demographics, and phone type, as well as to examine convergent validity with laboratory-based cognitive tests. Independent *t* tests, ANOVAs with follow-up Tukey HSD pairwise comparisons, and Pearson *r* correlations were used for dichotomous (eg, diagnostic group), >2-level categorical (eg, phone type), and continuous variables (eg, age), respectively.

Linear mixed effect regression models examined the practice effects for each mobile cognitive test to understand whether performance improved as a function of study day. Mixed effect Poisson regression was used for each mobile cognitive test outcome (ie, Odd One Out total score). An interaction with diagnostic status was initially included in each mixed effect model to examine whether practice effects differed by diagnostic status. If the interaction was not significant at *P*<.05, then diagnostic status and its interaction with the study day were removed from the model to simply estimate practice effects in the overall sample. When significant practice effects were identified, spline regression models were conducted to determine whether there was a point (study day) at which the performance stabilized. All statistical analyses were performed using R (version 3.5.0; R Foundation for Statistical Computing). Mixed effect models were conducted using the *lme4* package [46].

### Public Significance Statement

There are several limitations to traditional tests of cognitive abilities, including the time, cost, and accessibility of neuropsychological services. This study provides initial research support for 7 newly developed mobile cognitive tests that can be easily self-administered on personal smartphones in real-world environments.

## Results

### Sample Characteristics

Demographic and clinical characteristics by diagnostic status are shown in Table 3. The groups were comparable in terms of demographics; they did not significantly differ in age, sex, race, ethnicity, or years of education. Participants with BD were more likely than healthy volunteers to be unemployed (23/45, 51% vs 1/21, 5%, respectively) and have a lower income. In addition, compared with healthy volunteers, participants with BD had significantly greater depressive symptomology and lower NIH-TB-CB Fluid Cognition, executive functions, and episodic memory on laboratory-based neuropsychological tests. However, cognitive performance in the BD group was still within normal limits, and the average mania severity scores were within the mild range. A total of 41% (27/66) of the participants used personal iPhones, 32% (21/66) used personal Android phones, and 27% (18/66) used study iPhones.

**Table 3:** Demographics and clinical characteristics by bipolar disorder status (N=66).

Characteristics			Bipolar disorder group (n=45)		Healthy volunteer group (n=21)		Cohen *d*	Test statistics^a^	*P* value
			Value, mean (SD)	Range or %	Value, mean (SD)	Range or %			
**Demographics**									
	Age (years)		43 (12)	19-61	42 (14)	18-65	0.09	*t*_64_=−0.36	.72
	Sex (women)		30 (N/A^b^)	66.7	15 (N/A)	71.4	N/A	*X*^2^_1_=0.2	.70
	**Race, n (%)**						N/A	*X*^2^_3_=6.2	.11
		White	26 (N/A)	57.8	8 (N/A)	38.1			
		Black or African American	4 (N/A)	8.9	2 (N/A)	9.5			
		Asian	2 (N/A)	4.4	5 (N/A)	23.8			
		Other	13 (N/A)	28.9	6 (N/A)	28.6			
	Ethnicity (Hispanic or Latino), n (%)		8 (N/A)	N/A	3 (N/A)	14.3	N/A	*X*^2^_1_=0.2	.70
	Education (years), mean (SD)		14.89 (N/A)	2.54	15.52 (N/A)	2.73	0.25	*t*_64_=0.92	.36
	**Employment status, n (%)**						N/A	*X*^2^_2_=12.73	.005^c^
		Unemployed	22 (N/A)	48.9	1 (N/A)	4.8			
		In school	1 (N/A)	2.2	1 (N/A)	4.8			
		Part-time employment	9 (N/A)	20	6 (N/A)	28.6			
		Full-time employment	13 (N/A)	28.9	13 (N/A)	61.9			
	**Residential status, n (%)**						N/A	*X*^2^_2_=0.5	.79
		Independent, financially responsible	36 (N/A)	80	17 (N/A)	81			
		Independent, not financially responsible	8 (N/A)	17.8	4 (N/A)	19			
		Unsupervised residential facility	0 (N/A)	0	0 (N/A)	0			
		Supervised residential facility	1 (N/A)	2.2	0 (N/A)	0			
	**Income (US $), n (%)**						N/A	*X*^2^_2_=7.7	.02^d^
		<19,000	24 (N/A)	53.3	4 (N/A)	19			
		20,000 to 74,999	12 (N/A)	26.7	12 (N/A)	57.1			
		>75,000	9 (N/A)	20	5 (N/A)	23.8			
	**Smartphone used for study, n (%)**						N/A	*X*^2^_2_=6.4	.04^d^
		Personal iPhone	14 (N/A)	31.1	13 (N/A)	61.9			
		Personal Android	18 (N/A)	40	3 (N/A)	14.3			
		Study-loaned phone	13 (N/A)	28.9	5 (N/A)	23.8			
**Substance use and mood**									
	**Alcohol, n (%)**						N/A	*X*^2^_2_=4.2	.13
		Abstinent	21 (N/A)	46.7	6 (N/A)	28.6			
		Infrequent-moderate	21 (N/A)	46.7	15 (N/A)	71.4			
		Heavy or very heavy	3 (N/A)	6.7	0 (N/A)	0			
	**Cannabis, n (%)**						N/A	*X*^2^_2_=6.2	.10
		Current abuse	2 (N/A)	4.4	0 (N/A)	0			
		Current dependence	2 (N/A)	4.4	0 (N/A)	0			
		Former use disorder	7 (N/A)	15.6	0 (N/A)	0			
	Beck Depression Inventory-II, mean (SD)		14.69 (9.80)	N/A	2.71 (3.33)	N/A	1.64	*t*_64_*=*−7.3	<.001^c^
	Baseline YMRS^e^, mean (SD)		6.13 (5.32)	N/A	N/A	N/A	N/A	N/A	N/A
**Laboratory-based neuropsychological scores^f^, mean (SD)**									
	NIH-TB^g^ Total Cognition Score		101.53 (17.13)	N/A	107.38 (11.32)	N/A	0.40	*t*_64_=1.42	.16
	NIH-TB Crystallized Intelligence Score		104.58 (14.69)	N/A	104.95 (14.27)	N/A	0.03	*t*_64_=0.10	.92
	NIH-TB Fluid Cognition Score		98.07 (19.11)	N/A	107.48 (11.29)	N/A	0.60	*t*_64_=2.09	.04^d^
	NIH-TB Flanker Inhibitory Control and Attention Test		91.89 (15.17)	N/A	93.38 (10.32)	N/A	0.11	*t*_64_=0.47	.64
	NIH-TB Dimensional Change Card Sort Test		101.31 (20.25)	N/A	110.38 (11.86)	N/A	0.55	*t*_64_=2.28	.03^d^
	NIH-TB Pattern Comparison Processing Speed Test		103.53 (20.13)	N/A	109.57 (17.06)	N/A	0.32	*t*_64_=1.19	.24
	NIH-TB List Sorting Working Memory Test		97.84 (15.43)	N/A	105.10 (12.87)	N/A	0.51	*t*_64_=1.87	.07
	NIH-TB Oral Reading Recognition Test		101.84 (13.18)	N/A	102.48 (13.60)	N/A	0.05	*t*_64_=0.18	.86
	NIH-TB Picture Sequence Memory Test		99.42 (17.33)	N/A	107.43 (12.54)	N/A	0.53	*t*_64_=2.13	.04^d^
	D-KEFS^h^ Color-Word Interference Test		10.82 (3.35)	N/A	12.48 (1.44)	N/A	0.64	*t*_64_=2.80	.007^c^

^a^*t* tests for continuous variables and chi-square tests for dichotomous variables.

^b^N/A: not applicable.

^c^*P*<.01.

^d^*P*<.05.

^e^YMRS: Young Mania Rating Scale.

^f^Demographically adjusted standard scores from the National Institutes of Health Toolbox Cognition Battery unless otherwise noted.

^g^NIH-TB: National Institutes of Health Toolbox.

^h^D-KEFS: Delis-Kaplan Executive Function System.

### Overall Adherence

**Figure 1:**
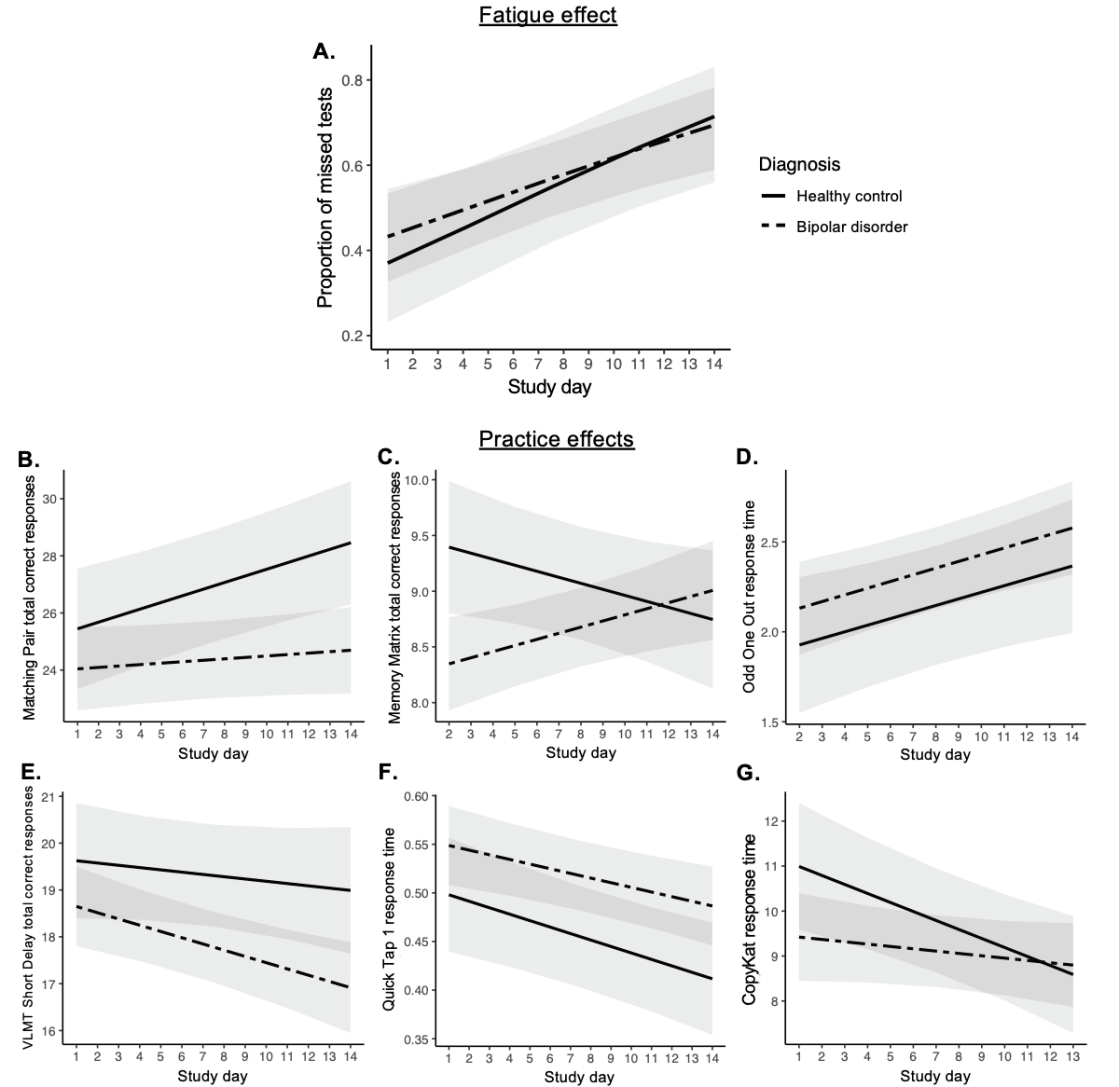
Fatigue effect and practice effects in mobile cognitive test completion over the 14-day study period. VLMT: Variable Difficulty List Memory Test.

Overall, the mean adherence to the EMCT protocol was 69.7% (SD 20.5%, range 28.6%-97.6%), resulting in 3965 valid and complete mobile cognitive tests among all 66 participants. Adherence did not differ by diagnostic status (*t*_64_=0.97; *P*=.33). In the overall sample, participants were more likely to miss a test as the number of study days increased (logit=0.093; odds ratio 1.100 [per 1-day increase], 95% CI 1.060-1.138; *P*<.001). This fatigue effect did not differ by diagnostic status (*P*=.45; Figure 1A).

### Mobile Cognitive Test Performance—Group, Demographic, and Phone Type Differences

Participants with BD were slower than the healthy volunteers on mean Quick Tap 1 performance; mean performance did not differ on the other mobile cognitive tests, although medium effect sizes were found for Matching Pair, Memory Matrix, and the VLMT (Table 4). In each case, the healthy volunteer group outperformed the BD group. Associations between mobile cognitive test performance and age, sex, education, and phone type are presented in Multimedia Appendix 3. In the full sample, age effects were found for Matching Pair, Memory Matrix, Odd One Out (response time), and Quick Tap 1 and 2; all findings were in the expected direction, with worse performance associated with older age. No sex effects were observed in this sample. Higher education was associated with better performance on all tests except Odd One Out (total score) and Quick Tap 2. Participants with personal iPhones had significantly higher scores on all tests (except Quick Tap 2) compared with participants with personal Androids or laboratory-given iPhones. Regarding ethnicity, there were no significant differences in mobile cognitive test performance between Hispanic or Latino and non-Hispanic or Latino participants with BD (*P*>.05 in all cases). Among healthy volunteers, non-Hispanic or Latino participants had a greater Odd One Out score (mean 8.47, SD 0.37) than Hispanic or Latino participants (mean 7.7, SD 0.90; *t*_19_=2.56; *P*=.02). Non-Hispanic or Latino healthy volunteers had a faster CopyKat reaction time (mean 9.33, SD 1.84) than Hispanic or Latino healthy volunteers (mean 12.09, SD 2.26; *t*_19_=−2.34; *P*=.03). There were no other significant differences in mobile cognitive test performance between Hispanic or Latino and non-Hispanic participants.

**Table 4:** Mobile cognitive test performance by bipolar disorder status (N=66).

Cognitive domain and individual tests		Bipolar disorder group (n=45), mean (SD; range)	Healthy volunteer group (n=21), mean (SD; range)	Cohen *d*	T-score	*P* value
**Processing speed**						
	Matching Pair (total score)	24.21 (5.23; 16.00-37.90)	26.72 (4.09; 18.67-34.60)	0.53	1.94	.06
	Odd One Out (response time)	2.27 (0.81; 1.15-5.59)	2.08 (0.50; 1.36-3.20)	0.28	−1.01	.32
	Quick Tap 1 (response time)	0.51 (0.14; 0.34-0.91)	0.45 (0.07; 0.35-0.65)	0.54	−1.89^a^	.02^b^
**Working memory**						
	Memory Matrix (total score)	8.59 (1.35; 5.50-11.00)	9.11 (0.82; 7.38-10.88)	0.47	1.92^a^	.06
	Odd One Out (total score)	8.2 (0.58; 5.67-8.89)	8.36 (0.51; 7.20-9.00)	0.29	1.08	.28
	CopyKat (total score)	9.08 (3.45; 2.60-19.33)	10.27 (2.52; 5.00-15.63)	0.39	1.41	.16
Recognition memory—VLMT^c^ (total score)		16.97 (2.35; 12.96-21.87)	18.2 (2.32; 12.79-23.37)	0.53	1.98	.05
Response inhibition—Quick Tap 2 (total score)		10.74 (1.14; 7.00-12.00)	11.03 (1.19; 6.33-11.89)	0.25	0.96	.34

^a^Levene test for equality of variances violated; equal variances were not assumed.

^b^*P*<.05.

^c^VLMT: Variable Difficulty List Memory Test.

### Mobile Cognitive Test Adherence and Performance

#### Overview

Correlations between average mobile cognitive test scores and laboratory-based cognitive performance are presented in Table 5 (BD participants) and Table 6 (healthy volunteers). The mobile cognitive tests were designed to measure fluid cognition (vs crystallized intelligence). In most cases, the average mobile cognitive test performance and composite score were moderately to strongly correlated with the validated NIH-TB-CB fluid measures (age-corrected Fluid Cognition Composite Score and individual test scores) and the D-KEFS Color-Word Interference Test (*P*<.001 in each case). Adherence, practice effects, convergent validity with the NIH-TB Fluid Cognition Composite Score and individual tests, convergent validity with the D-KEFS Color-Word Interference Test, and intraindividual variability are provided below.

**Table 5:** Correlations between mobile cognitive tests and in-laboratory neuropsychological performance in the bipolar disorder sample (N=45).

		NIH^a^ Toolbox Cognition Battery^b^							D-KEFS^c^ Color-Word Interference Test
		Fluid Cognition Composite Score	Flanker Inhibitory Control and Attention Test	Dimensional Change Card Sort Test	Pattern Comparison Processing Speed Test	List Sorting Working Memory Test	Picture Sequence Memory Test		
**Processing speed**									
	Matching Pair	0.637^d^	0.302^e^	0.488^d^	0.497^d^	0.518^d^	0.469^d^	0.592^d^	
	Odd One Out (response time)	−0.537^d^	−0.385^e^	−0.439^d^	−0.419^d^	−0.327^e^	−0.354^e^	−0.220	
	Quick Tap 1	−0.575^d^	−0.448^e^	−0.522^d^	−0.471^d^	−0.327^e^	−0.290	−0.364^d^	
**Working memory**									
	Memory Matrix	0.582^d^	0.217	0.509^d^	0.430^d^	0.495^d^	0.421^d^	0.431^d^	
	Odd One Out (total score)	0.154	−0.096	0.117	0.043	0.329^e^	0.168	0.285	
	CopyKat	0.537^d^	0.234	0.460^d^	0.408^d^	0.383^d^	0.419^d^	0.564^d^	
**Recognition memory**									
	VLMT^f^	0.213	−0.072	0.228	0.047	0.336^e^	0.224	0.535^d^	
**Response inhibition**									
	Quick Tap 2	−0.083	−0.178	−0.069	0.075	0.115	−0.251	0.335^e^	
Composite score		0.494^d^	0.072	0.379^e^	0.352^e^	0.526^d^	0.427^d^	0.664^d^	

^a^NIH: National Institutes of Health.

^b^Age-corrected standard scores; lower scores indicate a slower (worse) performance, and higher scores indicate a better performance.

^c^D-KEFS: Delis-Kaplan Executive Function System.

^d^*P*<.01.

^e^*P*<.05.

^f^VLMT: Variable Difficulty List Memory Test.

**Table 6:** Correlations between mobile cognitive tests and in-laboratory neuropsychological performance in the healthy control sample (N=21).

		NIH^a^ Toolbox Cognition Battery^b^						D-KEFS^c^ Color-Word Interference Test
		Fluid Cognition Composite Score	Flanker Inhibitory Control and Attention Test	Dimensional Change Card Sort Test	Pattern Comparison Processing Speed Test	List Sorting Working Memory Test	Picture Sequence Memory Test	
**Processing speed**								
	Matching Pair	0.483^d^	−0.08	−0.357	0.540^d^	0.641^e^	0.451^d^	−0.101
	Odd One Out (response time in seconds)	−0.317	0.076	0.286	−0.301	−0.406	−0.439^d^	0.355
	Quick Tap 1	−0.459^e^	−0.011	−0.048	−0.454^e^	−0.100	−0.600^e^	−0.081
**Working memory**								
	Memory Matrix	0.540^d^	0.077	−0.041	0.595^e^	0.38	0.394	0.077
	Odd One Out (total score)	0.496^d^	0.199	0.151	0.373	0.266	0.389	0.236
	CopyKat	0.376	−0.088	−0.025	0.411	0.349	0.314	0.178
**R** **ecognition memory**								
	VLMT^f^	0.428	−0.059	−0.024	0.277	0.535^d^	0.414	−0.345
**Response inhibition**								
	Quick Tap 2	0.001	−0.093	−0.15	0.205	0.067	−0.11	0.223
Composite score		0.478^d^	−0.021	0.175	0.511^d^	0.608^e^	0.292	0.015

^a^NIH: National Institutes of Health.

^b^Age-corrected standard scores; lower scores indicate slower (worse) performance, and higher scores indicate a better performance.

^c^D-KEFS: Delis-Kaplan Executive Function System.

^d^*P*<.05.

^e^*P*<.01.

^f^VLMT: Variable Difficulty List Memory Test.

#### Matching Pair

The mean adherence to Matching Pair was 75.3% (SD 23.7%, range 11.1%-100%), and adherence did not differ by diagnostic status (*t*_64_=0.96; *P*=.34). There was a significant difference in practice effect by diagnostic status (estimate=−0.18; SE 0.09; *P*=.04) such that scores increased within persons across days among healthy volunteers (estimate=0.23; SE 0.08; *P*=.003) but not among persons with BD (estimate=0.05; SE 0.05; *P*=.32; Figure 1B). Spline regressions did not identify any point at which Matching Pair performance stabilized among the healthy volunteers. In both groups, Matching Pair was moderately to strongly associated with the NIH-TB Fluid Cognition Composite Score (BD participants: *r*=0.64 and *P*<.001; healthy volunteer participants: *r*=0.48 and *P*=.03). Intraindividual variability did not differ by diagnostic status (*t*_64_=0.78; *P*=.44).

#### Memory Matrix

The mean adherence in the overall sample to Memory Matrix was 75.3% (SD 21.3%, range 22.2%-100%). Participants with BD had lower adherence than healthy volunteers (292/405, 72.1% vs 155/189, 82%), but the difference was not statistically significant (*t*_64_=1.79; *P*=.08). There was a significant difference in practice effect by diagnostic status (estimate=0.11; SE 0.04; *P*=.002) such that (in contrast to Matching Pair) scores increased within persons among those with BD (estimate=0.05; SE 0.02; *P*=.005) but not among healthy volunteers (estimate=−0.05; SE 0.03; *P*=.12; Figure 1C). Spline regression did not identify any point at which Memory Matrix performance stabilized in the BD group. Memory Matrix was strongly related to the NIH-TB Fluid Cognition Composite Score in both groups (BD participants: *r*=0.58 and *P*<.001; healthy volunteer participants: *r*=0.54 and *P*=.01). Intraindividual variability did not differ by diagnostic status (*t*_64_=0.54; *P*=.59).

#### Odd One Out

The mean adherence in the overall sample to the Odd One Out mobile cognitive test was 69.9% (SD 21.8%, range 11.1%-100%), and adherence did not differ by diagnostic status (*t*_64_=0.67; *P*=.51). For the Odd One Out total correct variable, there was no practice effect in the overall sample (*P*=.15), nor was there a difference by diagnostic status (*P*=.80). For the Odd One Out response time variable, participants’ average response times increased across days in the overall sample, indicating worsening performance over time (estimate=0.037; SE 0.01; *P*<.001). This effect did not differ by diagnostic status (*P*=.97). Item difficulty did not vary across Odd One Out trials. There was no difference in this effect by diagnostic status (*P*=.97; Figure 1D). Spline regressions identified that the Odd One Out average response time increased significantly until day 4 (the second remote administration; days 1-4 regression estimate=0.42; SE 0.05; *P*<.001), after which response times stabilized (days 4-14 regression estimate=−0.01; SE 0.01; *P*=.30). The Odd One Out total correct score was unrelated to the NIH-TB Fluid Cognition Composite Score in the BD group (*r*=0.15; *P*=.36), but these variables were strongly related in the healthy volunteer group (*r*=0.50; *P*=.02). Conversely, the Odd One Out response time was strongly associated with the NIH-TB Fluid Cognition Composite Score in the BD group (*r*=−0.54; *P*<.001), but these variables were unrelated in the healthy volunteer group (*r*=−0.32; *P*=.13). Intraindividual variability of Odd One Out total correct score (*t*_64_=0.57; *P*=.57) and response time (*t*_64_=−0.90; *P*=.37) did not differ by diagnostic status.

#### Mobile VLMT

The mean adherence to the VLMT short delay in the overall sample was 66.5% (SD 18.9%, range 18.2%-100%). Adherence did not differ by diagnostic status (*t*_64_=0.74; *P*=.46). For the VLMT long delay, the mean adherence was 63.5% (SD 27.6%, range 0%-100%), which again did not differ by diagnostic status (*t*_64_=−0.38; *P*=.70). In the overall sample, although there was no practice effect for the short delay, participants appeared to recall fewer total correct words at the short delay over time (estimate=−0.10; SE 0.03; *P*=.002). This effect did not differ by diagnostic status (*P*=.21; Figure 1E). Spline regressions identified that short-delay scores decreased significantly until day 7 (seventh remote administration; days 1-7 regression estimate=−0.13; SE 0.07; *P*=.05), after which the scores stabilized (days 7-14 regression estimate=−0.07; SE 0.07; *P*=.31). For the VLMT long delay, there was no significant practice effect for the VLMT total correct score in the overall sample (estimate=−0.03; SE 0.05; *P*=.61), nor did this effect differ by diagnostic status (*P*=.24). The VLMT was not significantly related to the NIH-TB Fluid Cognition Composite Score in either group (BD participants: *r*=0.21 and *P*=.16; healthy volunteer participants: *r*=0.43 and *P*=.05). This shows good discriminant validity given that the NIH-TB-CB does not include a verbal recognition test. Intraindividual variability did not differ by diagnostic status (*t*_64_=0.79; *P*=.44).

We also examined within-person forgetting on the VLMT. From short to long delay, participants lost an average of 3.8 words (SD 2.4, range −5 to 12; negative values reflect words gained from short to long delay). The average number of words lost from short to long delay was not related to diagnostic status (healthy volunteer mean 4.6 words, BD mean 3.4 words; *t*_62_=1.8; *P*=.07) or demographic characteristics of the participants, including age (*b*=−0.00; SE 0.025; *P*=.90), sex (*b*=−0.01; SE 0.65; *P*=.88), years of education (*b*=−0.02; SE 0.12; *P*=.89), ethnicity (Hispanic vs non-Hispanic; *b*=0.79; SE 0.80; *P*=.33), or White (vs other) race (*b*=−0.59; SE 0.60; *P*=.33).

#### Quick Tap 1

The mean adherence to Quick Tap 1 was 72.4% in the overall sample (SD 20.9%, range 22.2%-100%), and adherence did not differ by diagnostic status (*t*_64_=1.01; *P*=.32). In the overall sample, there was a slight but significant practice effect for Quick Tap 1 response time. Response times decreased within persons across days (estimate=−0.01; SE 0.00; *P*<.001). This effect did not differ by diagnostic status (*P*=.46; Figure 1F). Spline regressions identified that response times were stable from day 1 to day 3 (days 1-3 regression estimate=0.01; SE 0.01; *P*=.27) and then significantly decreased after day 3 (days 3-14 regression estimate=−0.01; SE 0.00; *P*<.001). Quick Tap 1 was strongly related to the NIH-TB Fluid Cognition Composite Score in both groups (BD participants: *r*=−0.58 and *P*<.001; healthy volunteer participants: *r*=−0.46 and *P*=.04). Intraindividual variability did not differ by diagnostic status (*t*_64_=−0.75; *P*=.46).

#### Quick Tap 2

Participants were 72.2% adherent to Quick Tap 2 on average (SD 21.1%, range 22.2%-100%); this did not differ by diagnostic status (*t*_64_=1.04; *P*=.30). In the overall sample, there was no practice effect for the Quick Tap 2 total correct response score (estimate=−0.00; SE 0.01; *P*=.76), and there was no difference in practice effect by diagnostic status (*P*=.63). Quick Tap 2 scores were unrelated to the NIH-TB Fluid Cognition Composite Score in both groups (BD participants: *r*=−0.08 and *P*=.59; healthy volunteer participants: *r*=0.001 and *P*=.99). Intraindividual variability did not differ by diagnostic status (*t*_64_=0.24; *P*=.81).

#### CopyKat

In the overall sample, participants were 77.9% adherent to CopyKat on average (SD 21.6%, range 22.2%-100%). Adherence did not differ by diagnostic status (*t*_64_=1.19; *P*=.24). In the overall sample, there was no practice effect for the CopyKat total correct response score (estimate=−0.07; SE 0.04; *P*=.13), and there was no difference by diagnostic status (*P*=.13). However, for CopyKat average reaction time, there was a significant difference in practice effect by diagnostic status (estimate=0.15; SE 0.07; *P*=.05) such that reaction time significantly decreased (ie, improved) among healthy volunteers over time (estimate=−0.20; SE 0.06; *P*=.001) but not among participants with BD (estimate=−0.05; SE 0.04; *P*=.24; Figure 1G). Spline regressions identified that, among healthy volunteers, average reaction times decreased significantly until day 9 performance (sixth administration; days 1-9 regression estimate=−0.20; SE 0.10; *P*=.05), after which reaction times stabilized (estimate=−0.20; SE 0.18; *P*=.27). The CopyKat total correct response score was strongly related to the NIH-TB Fluid Cognition Composite Score in BD participants (*r*=0.54; *P*<.001); these variables were unrelated in the healthy volunteer group (*r*=0.38; *P*=.09). Intraindividual variability for the CopyKat total correct response score did not differ by diagnostic status (*t*_64_=1.83; *P*=.08).

## Discussion

### Principal Findings

The findings of this study support the acceptability and preliminary psychometric properties of 7 brief, repeatable, newly developed mobile cognitive tests assessing processing speed, reaction time, visual working memory, recognition memory, and response inhibition in people with BD as well as in a small sample of healthy volunteers. The test stimuli can be used on personal smartphones and, thus, were designed with a simple user interface to accommodate a diversity of human-level factors (eg, varying cognitive abilities and range of sociodemographic groups) as well as a diversity of device factors (eg, different operating systems and screen sizes). Given that these tests are intended to be self-administered, a combination of clear verbal and visual instructions was incorporated. The main findings of this study, which were broadly consistent with our hypotheses, include (1) adequate adherence to the study protocol, with participants completing an average of 70% of the 42 EMCT administrations over a 14-day period, and no group differences in adherence; (2) differences in sociodemographic factors and mobile cognitive test performance based on ownership of an iPhone versus an Android device [47]; (3) moderate to strong correlations between the mobile cognitive tests and in-laboratory neuropsychological performance in the whole sample; (4) a fatigue effect such that participants were more likely to miss tests as the number of study days increased (with no differences between the BD and healthy volunteer groups); and (5) small practice effects, primarily among tests assessing response time. This study adds to the growing literature supporting the convergent and discriminant validity of mobile cognitive testing by demonstrating greater degrees of shared variance between mobile and laboratory-based tests of similar constructs than between mobile and laboratory-based tests of disparate constructs (Tables 4 and 5) [13,14,16,45,48].

The only test in which we found a statistically significant difference between the groups was Quick Tap 1. In both groups, low intraindividual variability was observed in Quick Tap 1; this test may be a good candidate for studies or clinical trials looking for a simple processing speed test that is not overly variable and may be most sensitive to cognitive dysfunction detection. Furthermore, the mobile cognitive tests that showed the most promise for testing real-world cognitive performance among people with BD (based on the examination of effect sizes) were tests tapping the domains of processing speed (Matching Pair and Quick Tap 1), working memory (Memory Matrix), and recognition memory (VLMT). These cognitive domains are associated with disability and mood state effects in persons with BD and, thus, may be useful tools for examining cognition change over time or response to treatment.

Of note, the correlations between our mobile cognitive tests and in-laboratory neuropsychological performance were less consistent in healthy controls than in participants with BD. This is likely related to the smaller sample size of the healthy control group and the more restricted range of scores on both mobile cognitive tests and in-laboratory neuropsychological tests among the healthy controls as limited variability restricts our ability to detect robust relationships between these measures. Future work should continue to validate these mobile tests in larger samples of healthy adults. Another finding we want to make note of is the unexpected results with the Odd One Out response times, in that participants did worse on this task (slower performance) as the number of study days increased. This could be due to fatigue or lower motivation to complete the task quickly after repeated administrations. Anecdotally, participants also reported to the study staff that they found this task “boring,” which may have affected test performance with repeated administrations. Nevertheless, adherence to this task was still high at an average of 69.9% (SD 21.8%), indicating acceptability among the participants. We also saw some evidence of ceiling effects for this task as well as Quick Tap 2, and further evaluation and iterations of these tasks are likely warranted to improve psychometrics and increase task engagement. Further work would also benefit from examining whether these tasks could be useful in other ways, such as measuring effort when completing fully remote ambulatory assessments.

This study adds to the limited literature on foundational psychometric research of an EMCT platform. Many of the mobile cognitive tests available for download do not have accompanying psychometric data to guide their use [49]. Previous studies support the feasibility and acceptability of mobile cognitive testing, with adherence rates ranging from approximately 79% to 90% in both clinical and nonclinical samples [14-16,45,50,51]. Most [16,48,52] but not all [13] previous investigations report small practice effects on selected tests, with no differences across clinical and nonclinical groups. A study reported a small impact of fatigue, with reductions in adherence rates across the 14-day procedure [16], whereas 2 other investigations reported no such fatigue effects [48,52].

### Limitations and Considerations for Future Research

This study is not without limitations. First, the sample size was relatively small, and the participants were of a specific psychiatric population, with only 32% (21/66) of healthy volunteer participants included. Our examination of intraindividual variability by diagnostic status was underpowered to detect meaningful differences; however, we included these data as they still provide valuable insights into performance in this sample. As previously mentioned, a recently published study provided validity data for the VLMT (6-, 12-, and 18-length word lists) among a large sample of persons with serious mental illness [15]. Further work is ongoing to continue the validation of these tasks and others in different populations, including larger groups of healthy volunteers and persons with mild cognitive impairment. We recommend that this ongoing (and future) work consider traditional sociodemographic factors that are known to affect cognitive test performance (age, sex, race or ethnicity, and education), the amount of variability in test scores that is attributable to device type, and digital literacy. Future work would also benefit from examining whether creating a composite variability index that includes performance on >1 test would yield variability data that are more sensitive than variability on each individual test.

Second, we were unable to examine the convergent validity of our tests with traditional laboratory-based cognitive domain scores, and the NIH-TB-CB does not include >1 test per domain to generate domain score data. However, we were able to demonstrate the overall construct validity of our mobile cognitive tests and our composite score (calculated using *Z* scores) with the NIH-TB Fluid Composite Score and present relationships with individual NIH-TB tests, providing proof of concept for future studies. Our sample size was insufficient to create demographically corrected T-scores with our mobile cognitive tests, which is the best-practice metric for creating composites [53]; subsequent studies are needed to generate large-scale normative data.

Third, although we examined smartphone type, we did not collect data on service providers (eg, T-Mobile and AT&T), all of which have different data speeds and connectivity, and we did not examine differences by screen size (eg, iPhone 8 vs iPhone 8 Plus), which may affect response times. Similarly, we did not collect smartphone metadata, which could create variance in test performance, such as accelerometry, touch sensitivity and latency (which, although improving in consistency, can differ by up to 100 milliseconds between different devices [54,55]), and frame rate for dynamic visual displays. Ongoing digital phenotyping studies by our group and several other research groups include the collection of both active (eg, surveys and mobile cognitive tests) and passive (eg, accelerometry, geolocation, keystroke data, and ambient noise levels) digital data, which can provide a more comprehensive picture of cognition in context as well as allow for the examination of the aforementioned limitations. Refer to Germine et al [34] for an excellent review of the challenges faced when digitizing neuropsychological testing as well as a road map of specific recommendations. In line with these recommendations, the approaches we have taken in this study include taking a user-centered design approach to developing tests and developing a flexible platform that can be modified to accommodate changes in technology and adapted or customized to individual investigator requirements.

A final limitation that applies to all remote mobile cognitive testing is that it is difficult to identify suspected cheating, such as whether the participant or someone else took the tests. One way to address this is to examine score distributions and flag outliers as potential instances of noncompliance. Other options would be to include the collection of biodata such as fingerprints or face IDs. Relatedly, it is difficult to assess effort in a mobile cognitive testing platform. Previous work has found a small effect of self-reported distractions and interruptions on mobile cognitive testing performance but also that convergent validity with laboratory-based tests was minimally affected by these factors [15]. Another potential indicator is a lack of a practice effect on a test in which a practice effect is expected.

### Conclusions

In conclusion, the 7 mobile cognitive tests we have presented in this study may serve as useful tools for brief, frequent ambulatory cognitive testing in a person’s everyday environment. Our data show that people with a well-characterized psychiatric disorder can and will complete self-administered mobile cognitive tests with good adherence. The tests are automatically scored, can be integrated with EMA surveys, and are available for other investigators to use; thereby, they are poised for further psychometric (including norming) studies and scalability. As the field of neuropsychology continues to migrate toward precision medicine, there are several advantages to the availability of psychometrically strong mobile cognitive tests, including complementing traditional neuropsychological assessments by gathering data outside of the controlled clinic environment, examining intraindividual variability and establishing more reliable estimates of cognitive performance over time, improving sensitivity to detect change and reducing the number needed to treat in clinical trials, and ultimately having the capability to detect brain dysfunction and risk of cognitive decline earlier than is possible with traditional assessment methods.

## Appendix

Multimedia Appendix 1 Skewness and kurtosis of aggregated mobile cognitive testing variables.

Multimedia Appendix 2 Intraclass correlation coefficient between participant groups.

Multimedia Appendix 3 Correlations among mobile cognitive tests, demographics, and phone type.

## Abbreviations

BD: bipolar disorder
C-SSRS: Columbia Suicide Severity Rating Scale
D-KEFS: Delis-Kaplan Executive Function System
EMA: ecological momentary assessment
EMCT: ecological momentary cognitive testing
NIH-TB: National Institutes of Health Toolbox
NIH-TB-CB: National Institutes of Health Toolbox Cognition Battery
VLMT: Variable Difficulty List Memory Test
