# Supplementary material for: Initial Psychometric Properties of 7 NeuroUX Remote Ecological Momentary Cognitive Tests Among People With Bipolar Disorder: Validation Study
Source: J Med Internet Res. 2022 Jul 29;24(7):e36665. doi: 10.2196/36665 (PMC9377465; doi:10.2196/36665)
Supplement: Multimedia Appendix 1 [file jmir_v24i7e36665_app1.docx]

**Supplemental Materials.**

**Table S1.**

*Skewness and Kurtosis of Aggregated Mobile Cognitive Testing Variables*

|  |  | Statistic | Std. Error |
| --- | --- | --- | --- |
| Matching Pair (Total Score) | Skewness | 0.16 | 0.295 |
|  | Kurtosis | -0.585 | 0.582 |
| Memory Matrix (Total Score) | Skewness | -0.497 | 0.295 |
|  | Kurtosis | -0.111 | 0.582 |
| Odd One Out (Total Score) | Skewness | -2.017 | 0.295 |
|  | Kurtosis | 6.099 | 0.582 |
| Odd One Out (Response Time) | Skewness | 1.797 | 0.295 |
|  | Kurtosis | 6.015 | 0.582 |
| Variable Difficulty List Memory Test | Skewness | 0.195 | 0.295 |
|  | Kurtosis | -0.571 | 0.582 |
| Quick Tap 1 (Response Time) | Skewness | 1.198 | 0.295 |
|  | Kurtosis | 1.388 | 0.582 |
| Quick Tap 2 (Total Score) | Skewness | -2.279 | 0.295 |
|  | Kurtosis | 5.703 | 0.582 |
| CopyKat (Total Score) | Skewness | 0.554 | 0.295 |
|  | Kurtosis | 0.625 | 0.582 |
