# Supplementary material for: Initial Psychometric Properties of 7 NeuroUX Remote Ecological Momentary Cognitive Tests Among People With Bipolar Disorder: Validation Study
Source: J Med Internet Res. 2022 Jul 29;24(7):e36665. doi: 10.2196/36665 (PMC9377465; doi:10.2196/36665)
Supplement: Multimedia Appendix 2 [file jmir_v24i7e36665_app2.docx]

**Table 2S.**

*Intraclass correlation coefficient (ICC) between participant groups*

|  | BD ICC | HC ICC | All Participants ICC |
| --- | --- | --- | --- |
| Matching Pair (Total Score) | 0.61 | 0.43 | 0.57 |
| Memory Matrix (Total Score) | 0.53 | 0.16 | 0.41 |
| Odd One Out (Total Score) | 0.25 | 0.17 | 0.22 |
| Odd One Out (Response Time) | 0.55 | 0.33 | 0.50 |
| Variable Difficulty List Memory Test (Total Score) | 0.28 | 0.28 | 0.29 |
| Quick Tap 1 (Response Time) | 0.61 | 0.33 | 0.57 |
| Quick Tap 2 (Total Score) | 0.43 | 0.44 | 0.43 |
| CopyKat (Total Score) | 0.46 | 0.25 | 0.40 |
