# Supplementary material for: Initial Psychometric Properties of 7 NeuroUX Remote Ecological Momentary Cognitive Tests Among People With Bipolar Disorder: Validation Study
Source: J Med Internet Res. 2022 Jul 29;24(7):e36665. doi: 10.2196/36665 (PMC9377465; doi:10.2196/36665)
Supplement: Multimedia Appendix 3 [file jmir_v24i7e36665_app3.docx]

**Table 3S.**

*Correlations between mobile cognitive tests, demographics, and phone type*

*Correlations between mobile cognitive test performance and age*

|  | Entire Sample; *r(p)* | Bipolar Disorder (*n*=45) | Healthy Volunteer (*n*=21) |
| --- | --- | --- | --- |
|  | Age (years) | | |
| Matching Pair (Total Score) | -0.419 (<.001)** | -0.507 (<.001)** | -0.257 (.262) |
| Memory Matrix (Total Score) | -0.370 (.002)** | -0.479 (<.001)** | -0.122 (.598) |
| Odd One Out (Total Score) | 0.235 (.057) | 0.118 (.441) | 0.505 (.020)* |

| CopyKat (Total Score) | 0.002 (.989) | -0.046 (.765) | 0.145 (.529) |
| --- | --- | --- | --- |
| VLMT (Total Score) | -0.064 (.610) | 0.130 (.396) | -0.386 (.084) |

| Odd One Out (Response Time in Sec.) | 0.569 (<.001)** | 0.554 (<.001)** | 0.718 (<.001)** |
| --- | --- | --- | --- |
| Quick Tap 1 (Response Time in Sec.) | 0.391 (.001)** | 0.482 (<.001)** | 0.206 (.370) |
| Quick Tap 2 (Total Score) | 0.268 (.030)* | 0.222 (.143) | 0.369 (.100) |

*T-tests comparing mobile cognitive test performance by sex*

|  | Sex (male/female) |
| --- | --- |

|  | Entire Sample; *t*(*p)* | Bipolar Disorder (*n*=45); *t*(*p)* | Healthy Volunteer (*n*=21); *t*(*p)* |
| --- | --- | --- | --- |

| Matching Pair (Total Score) | 0.27 (.785) | -0.24 (.813) | 0.88 (.390) |
| --- | --- | --- | --- |
| Memory Matrix (Total Score) | -0.43 (.665) | -0.77 (.443) | 0.54 (.595) |
| Odd One Out (Total Score) | -0.659 (.512) | -0.81 (.424) | -0.02 (.982) |

| CopyKat (Total Score) | -0.83 (.410) | -0.88 (.384) | -0.24 (.813) |
| --- | --- | --- | --- |
| VLMT (Total Score) | -0.24 (.813) | -0.84 (.406) | 0.67 (.512) |

| Odd One Out (Response Time in Sec.) | 0.35 (.729) | 1.02 (.312) | -1.53 (.142) |
| --- | --- | --- | --- |
| Quick Tap 1 (Response Time in Sec.) | 1.05 (.300) | 1.56 (.116) | -0.89 (.387) |
| Quick Tap 2 (Total Score) | -0.54 (.588) | -0.41 (.683) | -0.44 (.668) |

*Correlations between mobile cognitive test performance and education*

|  | Education (years) |
| --- | --- |

|  | Entire Sample; *r(p)* | Bipolar Disorder (*n*=45) | Healthy Volunteer (*n*=21) |
| --- | --- | --- | --- |

| Matching Pair (Total Score) | 0.454 (<.001)** | 0.451 (.002)** | 0.434 (.049)* |
| --- | --- | --- | --- |
| Memory Matrix (Total Score) | 0.495 (<.001)** | 0.511 (<.001)** | 0.459 (.037)* |
| Odd One Out (Total Score) | 0.225 (.070) | 0.154 (.312) | 0.351 (.119) |

| CopyKat (Total Score) | 0.515 (<.001)** | 0.585 (<.001)** | 0.312 (.168) |
| --- | --- | --- | --- |
| VLMT (Total Score) | 0.455 (<.001)** | 0.338 (.023)* | 0.662 (.001)** |

| Odd One Out (Response Time in Sec.) | -0.337 (.005)** | -0.369 (.013)* | -0.227 (.322) |
| --- | --- | --- | --- |
| Quick Tap 1 (Response Time in Sec.) | -0.467 (<.001)** | -0.526 (<.001)** | -0.377 (.092) |
| Quick Tap 2 (Total Score) | 0.089 (.477) | 0.125 (.413) | -0.019 (.935) |

*Correlations between mobile cognitive test performance and phone type*

|  | Phone Type Used in Study |
| --- | --- |

|  | Personal iPhone, M(SD)  (n=27) | Personal Android, M(SD)  (n=22) | Study Issued iPhone,  M(SD) (n=18) | Omnibus Group Difference |
| --- | --- | --- | --- | --- |

| Matching Pair (Total Score) | 27.8(4.6) | 23.1(4.3) | 22.8(4.7) | F(2,64)=9.16, *p*<0.001** |
| --- | --- | --- | --- | --- |
| Memory Matrix (Total Score) | 9.3(1.0) | 8.5(1.3) | 8.2(1.1) | F(2,63)=5.91, *p*=0.004** |
| Odd One Out (Total Score) | 8.4(0.4) | 8.0(0.7) | 8.3(0.5) | F(2,63)=3.43, *p*=0.039* |

| CopyKat (Total Score) | 10.8(2.8) | 7.5(2.5) | 9.8(3.5) | F(2,63)=7.53, *p*=0.001** |
| --- | --- | --- | --- | --- |
| VLMT-Short Delay  (Total Score) | 19.4(1.9) | 17.5(3.1) | 18.0(2.5) | F(2,63)=3.92, *p*=0.025* |

| Odd One Out  (Response Time in Sec.) | 1.9(0.5) | 2.2(0.7) | 2.7(0.9) | F(2,63)=6.91,  *p*=0.002** |
| --- | --- | --- | --- | --- |
| Quick Tap 1  (Response Time in Sec.) | 0.44(0.1) | 0.54(0.1) | 05(0.2) | F(2,63)=5.36,  *p*=0.007** |
| Quick Tap 2 (Total Score) | 10.9(1.1) | 10.6(1.4) | 11.0(1.0) | F(2,63)=0.57,  *p*=0.568 |

**p* < 0.05; ***p* < 0.01
